# Tick-borne pathogens in Finland: comparison of *Ixodes ricinus* and *I. persulcatus* in sympatric and parapatric areas

**DOI:** 10.1186/s13071-018-3131-y

**Published:** 2018-10-24

**Authors:** Maija Laaksonen, Tero Klemola, Eeva Feuth, Jani J. Sormunen, Anna Puisto, Satu Mäkelä, Ritva Penttinen, Kai Ruohomäki, Jari Hänninen, Ilari E. Sääksjärvi, Ilppo Vuorinen, Hein Sprong, Jukka Hytönen, Eero J. Vesterinen

**Affiliations:** 1 Department of Biology, University of Turku, Turku, Finland; 2 Institute of Biomedicine, University of Turku, Turku, Finland; 3 Biodiversity Unit, University of Turku, Turku, Finland; 4 Centre for Infectious Disease Control, National Institute for Public Health and Environment (RIVM), Bilthoven, Netherlands; 5 Department of Agricultural Sciences, University of Helsinki, Helsinki, Finland

**Keywords:** *Ixodes ricinus*, *Ixodes persulcatus*, *Borrelia burgdorferi*, *Rickettsia*, “*Candidatus* Neoehrlichia mikurensis”, *Babesia*, *Anaplasma*, Distribution, Sympatric, Parapatric

## Abstract

**Background:**

Almost 3500 tick samples, originally collected *via* a nationwide citizen science campaign in 2015, were screened to reveal the prevalence and distribution of a wide spectrum of established and putative tick-borne pathogens vectored by *Ixodes ricinus* and *I. persulcatus* in Finland. The unique geographical distribution of these two tick species in Finland allowed us to compare pathogen occurrence between an *I. ricinus-*dominated area (southern Finland), an *I. persulcatus-*dominated area (northern Finland), and a sympatric area (central Finland).

**Results:**

Of the analysed ticks, almost 30% carried at least one pathogen and 2% carried more than one pathogen. A higher overall prevalence of tick-borne pathogens was observed in *I. ricinus* than in *I. persulcatus*: 30.0% (604/2014) *versus* 24.0% (348/1451), respectively. In addition, *I. ricinus* were more frequently co-infected than *I. persulcatus*: 2.4% (49/2014) *versus* 0.8% (12/1451), respectively. Causative agents of Lyme borreliosis, i.e. bacterial genospecies in *Borrelia burgdorferi* (*sensu lato*) group, were the most prevalent pathogens (overall 17%). “*Candidatus* Rickettsia tarasevichiae” was found for the first time in *I. ricinus* ticks and in Finnish ticks in general. Moreover, *Babesia divergens*, *B. venatorum* and “*Candidatus* Neoehrlichia mikurensis” were reported for the first time from the Finnish mainland.

**Conclusions:**

The present study provides valuable information on the prevalence and geographical distribution of various tick-borne pathogens in *I. ricinus* and *I. persulcatus* ticks in Finland. Moreover, this comprehensive subset of ticks revealed the presence of rare and potentially dangerous pathogens. The highest prevalence of infected ticks was in the *I. ricinus*-dominated area in southern Finland, while the prevalence was essentially equal in sympatric and *I. persulcatus-*dominated areas. However, the highest infection rates for both species were in areas of their dominance, either in south or north Finland.

**Electronic supplementary material:**

The online version of this article (10.1186/s13071-018-3131-y) contains supplementary material, which is available to authorized users.

## Background

Ticks are recognized as the primary vectors for several pathogenic viruses, bacteria and protozoa worldwide [1, 2]. In northern Europe, most notable tick-borne pathogens are *Borrelia burgdorferi* (*sensu lato*) spirochetes, of which at least seven genospecies are responsible for causing Lyme borreliosis (LB) [3, 4]. In Finland, the primary vectors for tick-borne pathogens are *Ixodes ricinus* (Linnaeus, 1758) and *Ixodes persulcatus* (Schulze, 1930). The nationwide distribution of these two tick species was studied recently [5] showing *I. ricinus* dominance in southern Finland, a sympatric area in central Finland and *I. persulcatus* dominance in northern Finland. Tick distribution patterns may have an important role in the distribution and diversity of tick-borne pathogens as well.

Studies have shown that *I. ricinus* and *I. persulcatus* are potential vectors for many microorganisms in addition to *B. burgdorferi* (*s.l.*) [6, 7]. These include TBE-virus (TBEV) causing tick-borne encephalitis (TBE), *Anaplasma phagocytophilum* causing human granulocytic anaplasmosis (HGA) and tick-borne fever (TBF) [8, 9], species of the bacterial genus *Rickettsia* causing spotted fever and typhus [10], *B. miyamotoi* spirochete causing hard tick-borne relapsing fever [11], “*Candidatus* Neoehrlichia mikurensis” causing neoehrlichiosis [12], *Babesia* protozoans causing babesiosis in animals and humans [13], *Francisella tularensis* causing tularemia [14] and *Bartonella henselae* causing cat scratch disease, even though transmission of *B. henselae* by ticks has not been established [15, 16]. The first nationwide investigation on the distribution of *Ixodes* ticks infected with *B. burgdorferi* (*s.l.*), TBEV and *B. miyamotoi* was recently published [5]. Moreover, the first reports of *A. phagocytophilum* and *Rickettsia* spp. in Finnish *I. ricinus* ticks were published in 2016 [17, 18]. Regarding *Bartonella henselae* and *F. tularensis*, no ticks infected by these pathogens have been found in Finland.

Despite the presence of many potential pathogens in ticks in Finland, only a few tick-borne infections other than LB and TBE have been reported in humans. A single case of fatal babesiosis was described in a man with a rudimentary spleen, detected at their autopsy in 2004 [19]. Underlying reasons for the apparent discrepancy may be, for example, low pathogenicity of the putative pathogens, unclear clinical manifestations and unestablished diagnostic criteria of human infections, lack of awareness among health-care professionals of the emerging tick-borne diseases, and unavailable laboratory tests. On the other hand, the co-occurrence of several pathogens in ticks can lead to co-infections with different tick-borne pathogens in humans and animals [20–23]. Co-infections can alter the dynamics of pathogen transmission and pathogen interactions within a host animal, and increase the severity of manifestations in humans [23–25]. For example, *A. phagocytophilum* infects human neutrophils, modulates the immune response of the host and thereby increases susceptibility to other pathogens, including *B. burgdorferi* (*s.l.*) [26]. Thus, co-infections of tick-borne pathogens can have a significant impact on the disease manifestations making the diagnostics of these infections more challenging.

The aim of the present study was to map the major tick-borne pathogens circulating in ticks in Finland. The distribution of *I. ricinus* and *I. persulcatus* within the country creates an exceptional opportunity to study the potential differences in the prevalence of tick-borne pathogens in a sympatric area compared to areas dominated by a single tick species.

## Methods

### Origin of the samples

In 2015, citizens were asked to send ticks *via* postal mail to the University of Turku as a part of a tick collection campaign. This collection resulted in nearly 20,000 individual ticks received from all around Finland, up to the Arctic Circle. Detailed information about the collection, acquisition of the samples and tick identification has been described in a previous study [5]. Because that study also presented the occurrence of TBEV and *B. miyamotoi* in a subset of 2000 tick samples, these pathogens were not included in the present study. The presence of *B. burgdorferi* (*s.l.*) at the genospecies level was also described in the previous study [5]. However, *B. burgdorferi* (*s.l.*) is included in the present study since it is now identified to the genospecies level.

**Fig. 1:**
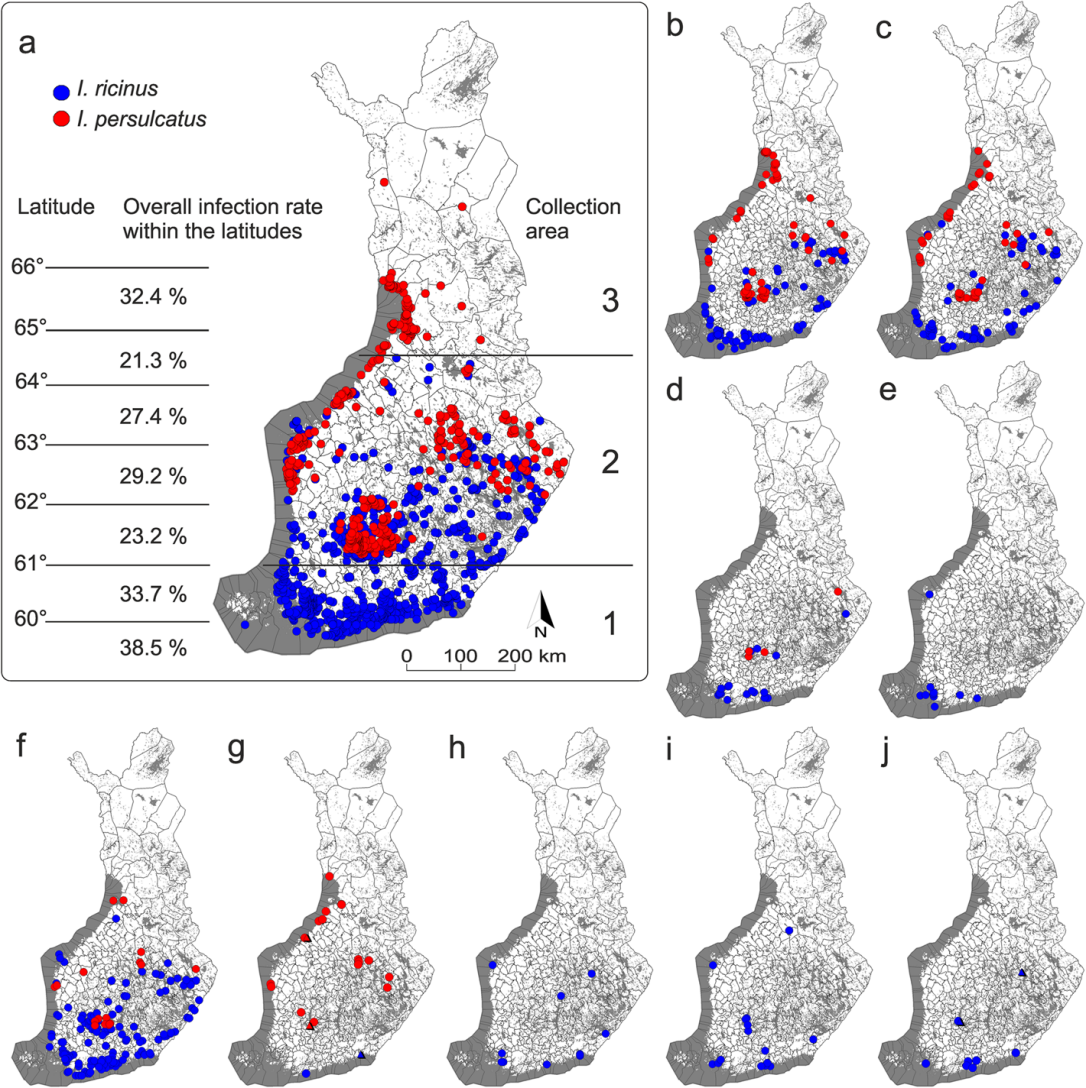
Geographical distribution of the analysed samples with collection information (coordinates) provided by citizens (*n* = 3418). Blue dots indicate collection points of *I. ricinus* samples and red dots *I. persulcatus*. **a** Map showing the geographical distribution of all samples analysed: *I. ricinus* (*n* = 1997) and *I. persulcatus* (*n* = 1421). Overall infection rates within the latitudes are shown in the left. 1: *I. ricinus*-dominated area; 2: sympatric area; and 3: *I. persulcatus-*dominated area are lined in the right. The following maps show the geographical distribution of the observed pathogens in the tick samples: **b** *Borrelia garinii* (*n* = 200); **c** *B. afzelii* (*n* = 149); **d** *B. valaisiana* (*n* = 25); **e** *B. burgdorferi* (*s.s.*) (*n* = 13); **f** *Rickettsia helvetica* (*n* = 231); **g** “*Candidatus* Rickettsia tarasevichiae” (*n* = 20) and *R. monacensis* (*n* = 3) as triangles; **h** *Anaplasma phagocytophilum* (*n* = 12); **i** “*Candidatus* Neoehrlichia mikurensis” (*n* = 17); **j** *Babesia venatorum* (*n* = 7) and *B. divergens* (*n* = 2) as triangles

A subset of 3465 ticks (2014 *I. ricinus* and 1451 *I. persulcatus*) out of a total of 20,000 ticks were screened for *B. burgdorferi* (*s.l.*), *Rickettsia* spp., *Babesia* spp., *Bartonella* spp., *Anaplasma* spp., *F. tularensis* and “*Ca.* N. mikurensis”. Of the 3465 samples, 175 were nymphs, four were larvae and the remaining were adults. The samples were selected to roughly represent both tick species (*I. ricinus* and *I. persulcatus*), and also the major collection areas, tick life stages and sex distribution of the whole collection [5]. Samples were further divided into three different distribution regions: 1, *I. ricinus-*dominated area (southern Finland); 2, sympatric area (central Finland) and 3, *I. persulcatus-*dominated area (northern Finland) (Fig. 1a).

DNA was extracted from the tick samples using NucleoSpin® RNA kits and RNA/DNA buffer sets (Thermo Fisher Scientific, Waltham, USA), following the kit protocols (RNA Kit: Rev. 16 May 2014 and RNA/DNA buffer set: Rev. 08 May 2014). DNA extracts were stored at -20 °C.

### Tick species identification using genetic methods

Tick species, if unknown after morphological identification (*n* = 146), was determined (decisively 95 *I. ricinus* and 51 *I. persulcatus*) in a species-specific duplex real-time quantitative PCR (qPCR) assay as previously described [18]. Briefly, IXO-I2-F4 and IXO-I2-R4 primers targeting a fragment of *Ixodes* spp. internal transcribed spacer 2 (ITS2) gene were used to amplify genus-specific segments, and Ipe-I2-P4 and Iri-I2-P4 probes were used to match the ITS2 region for either tick species (*I. persulcatus* or *I. ricinus*, respectively; Table 1). DNA samples from *I. ricinus* and *I. persulcatus* confirmed by sequencing in an earlier study [27] were used as positive controls, and double-distilled water (ddH_2_O) was used instead of sample DNA as a negative control in each assay.

**Table 1:** Primers and probes used in tick species determination and pathogen screening

Primer/probe name	Target	Nucleotide sequence (5' → 3')	Amplicon size (bp)^a^	Reference
Real-time: PCR				
Bbsl-ospA-F	*B. burgdorferi ospA*	AATATTTATTGGGAATAGGTCTAA	59	[83]
Bbsl-ospA-R		CACCAGGCAAATCTACTGA		
Bbsl-ospA-P		[6FAM]-TTAATAGCATGTAAGCAAAATGTTAGCA-[DDQ1]		
IXO-I2-F4	*Ixodes* spp. ITS2	TCTCGTGGCGTTGATTTGC	64	[27]
IXO-I2-R4	*Ixodes* spp. ITS2	CTGACGGAAGGCTACGACG		
Ipe-I2-P4	*I. persulcatus* ITS2	[FAM]-TGCGTGGAAAGAAAACGAG-[BHQ1]		
Iri-I2-P4	*I. ricinus* ITS2	[VIC]-TGCTCGAAGGAGAGAACGA-[BHQ1]		
Rspp-F	*Rickettsia* spp. *gltA*	GAGAGAAAATTATATCCAAATGTTGAT	100	[84]
Rspp-R		AGGGTCTTCGTGCATTTCTT		
Rspp-P		[Cy5]-CATTGTGCCATCCAGCCTACGGT-[BHQ3]		
Bab18S-F	*Babesia* spp. *18S* rRNA	CAGCTTGACGGTAGGGTATTGG	20	[85]
Bab18S-R		TCGAACCCTAATTCCCCGTTA		
Bab18S-P		[HEX]-CGAGGCAGCAACGG-[BHQ1]		
ApMSP2-F	*Anaplasma* spp. *Msp2*	ATGGAAGGTAGTGTTGGTTATGGTATT	30	[86]
ApMSP2-R		TTGGTCTTGAAGCGCTCGTA		
ApMSP-P		[CY5]-TGGTGCCAGGGTTGAGCTTGAGATTG-[BBQ650]		
CNeGroEL-F	“*Ca.* Neoehrlichia mikurensis” *GroEL*	CCTTGAAAATATAGCAAGATCAGGTAG	47	[87]
CNeGroEL-R		CCACCACGTAACTTATTTAGCACTAAAG		
CNeGroEL-P		[FAM]-CCTCTACTAATTATTGCWGAAGATGTAGAAGG TGAAGC-[BHQ1]		
BartssRA-F	*Bartonella* spp. ssrA	GCTATGGTAATAAATGGACAATGAAATAA	255	[88]
BartssRA-R		GCTTCTGTTGCCAGGTG		
BartssRA-P		[FAM]-ACCCCGCTTAAACCTGCGACG-[BHQ1]		
Ftu23-F	*F. tularensis* 23 *Kda*	TGAGATGATAACAAGACAACAGGTAAC	30	[89]
Ftu23-R		GGATGAGATCCTATACATGCAGTAGGA		
Ftu23-P		[FAM]-CCATTCATGTGAGAACTG-[BHQ1]		
PCR				
B5Sborseq-F	*Borrelia* IGS	GAGTTCGCGGGAGAGTAGGTTATTGCC	367	[28]
23Sborseq-R		TCAGGGTACTTAGATGGTTCACTTCC		
CS877-F	*Rickettsia* spp. *gltA*	GGGGACCTGCTCACGGCGG	381	[90]
CS1258-R		ATTGCAAAAAGTACAGTGAACA		
Ana2-F	*Anaplasma* spp. *16S* rRNA	CAAGCTTAACACATGCAAGTCGAAC	894	[91]
Ana2-R		CCCTTCCGTTAAGAAGGATCTAATC		
BabNu2-F	*Babesia* spp. *18S*	GACACAGGGAGGTAGTGACAAG	357	[92]
BabNu2-R		CTAAGAATTTCACCTCTGACAGT		

*Abbreviations*: *ospA* outer surface protein, *ITS2* internal transcribed spacer, *gltA* bacterial citrate synthase gene, *18S and 16S* ribosomal RNA genes, *Msp2* surface protein antigen, *GroEL* chaperonin protein, *ssrA* transfer-messenger RNA, *Kda* lipoprotein, *IGS* intergenic spacer region

^a^Amplicon size without nucleotides of primers

### Detection of pathogens in tick samples

Bbsl-ospA-F and Bbsl-ospA-R primers, and a Bbsl-ospA-P probe (Table 1) amplifying a fragment of the outer surface protein A (*ospA*) gene, were used to detect *B. burgdorferi* (*s.l.*) DNA, as previously described [5]. Positive and negative controls [*B. burgdorferi* (*sensu stricto*) strain B31 ATCC 35210 and ddH_2_O, respectively] were included in all runs.

For screening *Rickettsia*, *Anaplasma*, “*Ca.* N. mikurensis”, *Babesia*, *F. tularensis* and *Bartonella*, aliquots of original DNA samples were first pooled (10 samples per pool, 5 μl of each sample) due to low expected prevalence. For *Anaplasma*, *Babesia* and “*Ca.* N. mikurensis” screening, multiplex qPCR assays were first used. Briefly, qPCRs were first performed in 11 μl reaction volumes using the primers and probes displayed in Table 1. For *Bartonella* and *Rickettsia* screening, a duplex qPCR assay was first used in 8 μl volume using the primers and probes displayed in Table 1. Primers and probe (Table 1) targeting the 23 KDa gene were used to detect *Francisella tularensis* DNA. Individual samples from positive pools were analysed in 5 μl reaction volume. Detailed protocols of all qPCR assays are presented in Additional file 1: Methods.

Samples were analysed in three replicate reactions carried out on 384-well plates. Three positive and negative control reactions were used in each assay. Samples were considered positive when successful amplification was detected in at least two replicate reactions. The thermal cycling profile used for all qPCR assays was: 95 °C for 5 min, followed by 50 cycles of 95 °C for 10 s and 60 °C for 30 s. Thermal cycling was carried out at the Finnish Microarray and Sequencing Centre (FMSC, Turku, Finland) using QuantStudio™ 12 K Flex Real-Time PCR System (Life Technologies Inc., Carlsbad, CA, USA). Results were analysed using QuantStudio™ 12 K Flex Software v.1.2.2.

### Sequencing

The 5S-23S rDNA (rrfA-rrlB) intergenic spacer region (IGS) was sequenced from samples found positive for *B. burgdorferi* (*s.l.*) to identify the bacteria to genospecies level as previously described [28].

Samples found positive for *Rickettsia* spp., *Anaplasma* spp. and *Babesia* spp. by qPCR were sequenced using PCR primers displayed in Table 1. Detailed protocols of all PCRs are presented in Additional file 1: Methods.

Electrophoresis was carried out to confirm amplification success by running 1 μl of PCR product on 1.5% agarose gel. PCR products were purified by mixing 1 μl EXO I enzyme, 1 μl SAP enzyme and 8 μl of PCR product, after which the samples were first incubated for 5 min at 37 °C and then heated for 10 min at 80 °C. Purified samples were sent to Macrogen Inc. Europe (Amsterdam, Netherlands) for sequencing. The sequences were trimmed using Geneious 11.1.2 and run through BLAST (www.ncbi.nlm.nih.gov/BLAST/) and compared with reference sequences listed in the GenBank nucleotide sequence database (www.ncbi.nlm.nih.gov/genbank/).

### Data management and statistical analyses

Some of the tick samples received in the collection campaign were delivered in one shipment, indicating that the samples were from one sender. Such ticks were not necessarily independent observations, as generally assumed by basic statistical tests, because they likely shared either collection location or host animal/human individual, or both. It was often the case that ticks in one letter represented only one tick species and developmental stage. Therefore, we conducted formal statistical testing only for a couple of specific cases (see below), but otherwise tabulated frequencies and percentage values in a descriptive manner only.

With statistical tests we specifically analysed whether there was a difference in total infection rate (all pathogens combined) and *B. burgdorferi* (*s.l.*) or *Rickettsia* spp. prevalence between adult *I. persulcatus* and *I. ricinus.* Other pathogen groups were not statistically analysed due to low infection prevalence. In the subsequent phase, we tested whether there was a difference in pathogen prevalence between *I. ricinus-* and *I. persulcatus-*dominated areas and the sympatric area. Larvae and nymphs were ignored due to their relatively low sample sizes (*n* = 179) and low numbers of positive pathogen detections. We modelled the probability of a tick sample being positive for a pathogen by running a generalized estimating equation (GEE), a specific type of generalized linear mixed model for clustered observations, with binomial error distribution and logit link function. The shipment ID was set as a clustering factor, while the species and collection area of the tick were fixed explanatory factors in consecutive tests but never entered as fixed factors to the same model. Statistical testing was run with the IBM SPSS Statistics software v.23 (Armonk, NY, USA).

## Results

### Overall pathogen prevalence

A total of 3465 tick samples, consisting of 2014 *I. ricinus* and 1451 *I. persulcatus* samples from the *I. ricinus-* and *I. persulcatus-*dominated areas and from the sympatric area, were analysed for the presence of pathogens, including the most common and some putative tick-borne pathogens circulating in ticks in Europe. Both the total infection rate and the diversity of different tick-borne pathogens was higher for *I. ricinus* (30.0%, five pathogen groups) than for *I. persulcatus* (24.0%, three pathogen groups) (Table 2). The GEE model conducted for the adult samples indicated significantly higher probability of finding infected *I. ricinus* [estimated marginal mean (with 95% confidence interval) was 0.31 (0.27–0.34)] than *I. persulcatus* [0.25 (0.22–0.29)] (Wald statistics, *χ*^2^ = 4.75, *df* = 1, *P* = 0.029). The total prevalence of mono- and co-infected ticks reached 25.7% (892/3465) and 1.7% (60/3465), respectively.

**Table 2:** Prevalence (%) of studied pathogens in *I. ricinus* and *I. persulcatus* samples

Species	Collection area	No. of ticks analysed	No. of ticks infected (%)	*B*. *burgdorferi* (*s.l.*) *n* (%)	*Rickettsia* spp. *n* (%)	“*Ca*. N. mikurensis” *n* (%)	*Anaplasma* spp. *n* (%)	*Babesia* spp. *n* (%)
*I. ricinus*	1	994	325 (32.6)	202 (20.3)	126 (12.6)	11 (1.1)	12 (1.2)	7 (0.7)
	2	998	277 (27.8)	124 (12.4)	152 (15.2)	6 (0.6)	7 (0.7)	4 (0.4)
	3	5	0	0	0	0	0	0
	a	17	2	1	1	0	0	0
Subtotal		2014	604 (30.0)	327 (16.2)	279 (13.9)	17 (0.8)	19 (1.0)	11 (0.5)
*I. persulcatus*	1	0	0	0	0	0	0	0
	2	1160	271 (23.4)	200 (17.2)	78 (6.7)	0	2 (0.2)	0
	3	261	69 (26.4)	55 (21.1)	16 (6.1)	0	0	0
	a	30	8	8	0	0	0	0
Subtotal		1451	348 (24.0)	263 (18.1)	94 (6.5)	0	2 (0.1)	0
Total		3465	952 (27.4)	590 (17.0)	373 (10.8)	17 (0.5)	21 (0.6)	11 (0.3)

*Abbreviations*: 1, *I. ricinus*-dominated area in south Finland; 2, sympatric area of both species in middle Finland; 3, *I. persulcatus-*dominated area in north Finland (Fig. 1a)

^a^Tick samples that were not categorized into collection areas due to inaccurate collection information provided by citizens

### Pathogen prevalence in parapatric and sympatric areas

The highest prevalence of infected ticks was observed in the *I. ricinus*-dominated area in southern Finland (32.6%), and the lowest prevalence in the sympatric area in central Finland (25.4%). The GEE model also indicated a significantly higher probability of finding infected adults from the *I. ricinus*-dominated area compared to the sympatric area [*I. ricinus*-dominated area, 0.31 (0.27–0.35); *I. persulcatus*-dominated area, 0.28 (0.22–0.36); sympatric area, 0.25 (0.23–0.27)] (*χ*^2^ = 7.01, *df* = 2, *P* = 0.030). However, the only pathogen group with significant difference in infection prevalence in adult samples between *I. ricinus*-dominated area and sympatric area was *B. burgdorferi* (*s.l.*) [*I. ricinus*-dominated area, 0.24 (0.20–0.29); *I. persulcatus*-dominated area, 0.18 (0.13–0.24); sympatric area, 0.14 (0.13–0.16)] (*χ*^2^ = 23.40, *df* = 2, *P* < 0.001).

When investigating the number of infected ticks on the different latitudes in steps of one degree (approximately 110 km), the highest infection rates were found from the area below the latitude of 60°N (38.5%, *n* = 104), between the latitudes of 60°N and 61°N (33.7%, *n* = 808) and from the area between the latitudes of 65°N and 66°N (32.4%, *n* = 148) (Fig. 1a). In contrast, the lowest rate was from the area between the latitudes of 64°N and 65°N (21.3%, *n* = 127).

### *B. burgdorferi* (*s.l.*) positivity

The most prevalent pathogen group was *B. burgdorferi* (*s.l.*), which was detected in 17.0% of the screened tick samples (Table 2). The prevalence was 16.2% for *I. ricinus* and 18.1% for *I. persulcatus*. A significantly higher probability of *B. burgdorferi* (*s.l.*) infection was found in *I. persulcatus* adults [0.22 (0.19–0.25)] compared with *I. ricinus* adults [0.16 (0.13–0.18)] (*χ*^2^ = 8.50, *df* = 1, *P* = 0.004), even in the sympatric area (*I. persulcatus* adults [0.17 (0.15–0.20)], *I. ricinus* adults [0.12 (0.10–0.15)], Wald statistics, *χ*^2^ = 7.68, *df* = 1, *P* = 0.006).

Out of the *B. burgdorferi* (*s.l.*) positive samples (*n* = 590), 394 were further identified to genospecies level (Table 3). Four different genospecies were identified. Sequences were at least 98% identical to the 5S-23S ribosomal RNA intergenic spacer of *B. valaisiana* (GenBank: KX906937 and KX906938 from Slovakia), *B. afzelii* (GQ387036 from Switzerland, KX906945 and KX906933 from Slovakia and KY038873 from Romania), *B. garinii* (KX906934, KX906935 and KX906940 from Slovakia) and *B. burgdorferi* (*s.s.*) (X57791, origin not given). Our sequences are presented in Additional file 2: Table S1. Among *I. ricinus* samples, *B. garinii* (44.6%) and *B. afzelii* (40.7%) were the predominant genospecies, followed by *B. valaisiana* (9.1%) and *B. burgdorferi* (*s.s.*) (5.6%). Among *I. persulcatus* samples, *B. garinii* was clearly the predominant genospecies (62.6%), followed by *B. afzelii* (35.0%) and *B. valaisiana* (2.5%). *B. burgdorferi* (*s.s.*) was not detected in *I. persulcatus*.

**Table 3:** Prevalence (%) of pathogen species identified by sequencing in *I. ricinus* and *I. persulcatus* samples

Species	No. (%) of ticks infected with certain pathogen species									
	Bg	Ba	Bv	Bss	Rh	CRt	Rm	Ap	Bad	Bav
*I. ricinus*	103/231 (44.6)	94/231 (40.7)	21/231 (9.1)	13/231 (5.6)	199/201 (99.0)	1/201 (0.5)	1/201 (0.5)	12/12 (100)	2/9 (22.2)	7/9 (77.8)
*I. persulcatus*	102/163 (62.6)	57/163 (35.0)	4/163 (2.5)	0	32/53 (60.4)	19/53 (35.8)	2/53 (3.8)	0	0	0
Total	205/394 (52.0)	151/394 (38.3)	25/394 (6.4)	13/394 (3.3)	231/254 (90.9)	20/254 (7.9)	3/254 (1.2)	12/12 (100)	2/9 (22.2)	7/9 (77.8)

*Abbreviations*: Bg, *Borrelia garinii*; Ba, *B. afzelii*; Bv, *B. valaisiana*; Bss, *B. burgdorferi* (*sensu stricto*); Rh, *Rickettsia helvetica*; CRt, “*Ca. R. tarasevichiae*”; Rm, *R. monacensis*; Ap, *Anaplasma phagocytophilum*; Bad, *Babesia divergens*; Bav, *B. venatorum*

The distribution maps drawn from the positive *B. burgdorferi* (*s.l.*) samples are shown in Fig. 1b-e. The highest prevalence of infected *I. ricinus* was observed in southern Finland (*I. ricinus*-dominated area), while the highest prevalence of infected *I. persulcatus* was observed in northern Finland (*I. persulcatus-*dominated area) (Table 2).

### *Rickettsia* spp. positivity

The next most prevalent pathogen group was *Rickettsia* spp., which was detected in 10.8% of the screened tick samples (Table 2). The prevalence was 13.9% for *I. ricinus* and 6.5% for *I. persulcatus*. The GEE model also indicated a significantly higher probability of finding *Rickettsia-*positive *I. ricinus* adults [0.13 (0.10–0.17)] than *I. persulcatus* adults [0.05 (0.04–0.08)] (*χ*^2^ = 27.17, *df* = 1, *P* < 0.001). Of the 373 positive samples, 254 were successfully sequenced (Table 3). From these, 231 amplicons were at least 98% identical to the *gltA* of *R. helvetica* (GenBank: KF447530 from France), 20 amplicons had at least 99% sequence identity with the *gltA* of “*Ca.* R. tarasevichiae” (KU361212 from Mongolia) and three amplicons were at least 98% identical to the *gltA* of *R. monacensis* (KY203388 from Italy). *Rickettsia helvetica* was clearly the most abundant species among *I. ricinus* samples (99%), and only two samples were positive for *R. monacensis* or “*Ca.* R. tarasevichiae” (Table 3). Among *I. persulcatus* samples, *R. helvetica* was also predominant (60.4%), followed by “*Ca.* R. tarasevichiae” (35.8%) and *R. monacensis* (3.8%). The distribution of the positive *Rickettsia* samples corresponded to the distribution of the whole subset of ticks (Fig. 1f, g).

### *Anaplasma* spp. positivity

In total, *Anaplasma* spp. was detected in 0.6% of the screened DNA samples (Table 2) The prevalence was 1.0% for *I. ricinus* and 0.1% for *I. persulcatus*. From 19 positive *I. ricinus* samples, 12 were identified as *A. phagocytophilum* by sequencing (Table 3). Amplicons were at least 99% identical to the *16S* of *A. phagocytophilum* (GenBank: KY114936 from Croatia). Neither of the two positive *I. persulcatus* samples could be identified to species level due to a poor DNA sequence trace. The distribution map drawn from the positive *A. phagocytophilum* samples is shown in Fig. 1h.

### Other pathogens

“*Ca.* N. mikurensis” was detected in 0.5% of the screened DNA samples and the prevalence was 0.8% for *I. ricinus* (Table 2). “*Ca.* N. mikurensis” DNA was not detected in *I. persulcatus* samples. The distribution of the positive “*Ca.* N. mikurensis” was rather aggregated as most of the positive samples were collected from urbanized areas near the cities of Helsinki, Tampere and Turku in southern Finland (Fig. 1i).

*Babesia* spp. was detected in 0.3% of all the screened DNA samples (Table 2), and the prevalence was 0.5% for *I. ricinus*. No infected *I. persulcatus* ticks were found. Nine positive samples were successfully sequenced, of which seven were identified as *B. venatorum* (77.8%) and two were identified as *B. divergens* (22.2%). Sequences were at least 99% identical to the reference sequences obtained from GenBank (*B. divergens*: U16370, origin not given, KY242392 from Poland; *B. venatorum*: KM289158 from Spain). The distribution of the positive *Babesia* samples was also rather aggregated (Fig. 1j). All positive samples from southern Finland were *B. venatorum* while *B. divergens* were found only from samples collected in central Finland.

*Francisella tularensis* and *Bartonella* spp. were not detected in either of the tick species.

### Co-infection of pathogens

Among all analysed ticks, 1.7% were found to be co-infected (Table 4). *Ixodes ricinus* were more frequently co-infected than *I. persulcatus*: 2.4 *vs* 0.8%, respectively. However, when investigating co-infections among infected adult ticks, no significant differences were observed between species or the three collection areas (species: *χ*^2^ = 1.50, *df* = 1, *P* = 0.221; collection areas: *χ*^2^ = 3.80, *df* = 2, *P* = 0.149). Co-infection prevalence for adult infected ticks was 8.1% for *I. ricinus* and 3.2% for *I. persulcatus*. A higher diversity of different pathogen infections was also observed in *I. ricinus* (6 combinations) than in *I. persulcatus* (1 combination). Most of the co-infections (68.3%) were between *B. burgdorferi* (*s.l.*) and *Rickettsia* spp. pathogens.

**Table 4:** Co-infection prevalence (%) between studied pathogens in *I. ricinus* and *I. persulcatus* samples

Species	Collection area	No. of ticks analysed	No. (%) of ticks co-infected	*B*. *b.* (*s.l.*) + *Rickettsia* spp.	*B. b.* (*s.l.*) + *Anaplasma spp.*	*B. b.* (*s.l.*) + *Babesia* spp.	*B. b.* (*s.l.*) + “*Ca.* N. mikurensis”	*Babesia* spp. + *Rickettsia* spp.	*Anaplasma* spp. + *Rickettsia* spp.
*I. ricinus*	1	994	33 (3.2)	17 (1.6)	2 (0.2)	3 (0.3)	6 (0.6)	1 (0.1)	4 (0.4)
	2	998	16 (1.6)	13 (1.3)	1 (0.1)	0	0	2 (0.2)	0
	3	5	0	0	0	0	0	0	0
	a	17	0	0	0	0	0	0	0
Subtotal		2014	49 (2.4)	30 (1.5)	3 (0.2)	3 (0.2)	6 (0.3)	3 (0.2)	4 (0.2)
*I. persulcatus*	1	0	0	0	0	0	0	0	0
	2	1160	9 (0.8)	9 (0.8)	0	0	0	0	0
	3	261	2 (0.8)	2 (0.8)	0	0	0	0	0
	a	30	0	0	0	0	0	0	0
Subtotal		1451	11 (0.8)	11 (0.8)	0	0	0	0	0
Total		3465	60 (1.7)	41 (1.2)	3 (0.1)	3 (0.1)	6 (0.2)	3 (0.1)	4 (0.1)

*Abbreviations*: *B.b.* (*s.l.*), *Borrelia burgdorferi* (*s.l.*); 1, *I. ricinus*-dominated area in south Finland; 2, sympatric area of both species in middle Finland; 3, *I. persulcatus*-dominated area in north Finland (Fig. 1a)

^a^Tick samples that were not categorized into collection areas due to inaccurate collection information provided by citizens

### Pathogens in larvae and nymphs

From 179 samples of juvenile life stages (nymph and larva), 33 (18.4%) were infected with *B. burgdorferi* (*s.l.*), *Rickettsia* or “*Ca.* N. mikurensis”. *Borrelia burgdorferi* (*s.l.*) and *Rickettsia* prevalences in juvenile life stages (13.4 and 5.6%, respectively) were lower than in adults (17.3 and 11.1%, respectively). In contrast, the prevalence of “*Ca.* N. mikurensis” in juvenile life stages was higher than in adults (2.2 *vs* 0.4%, respectively). However, three of the four “*Ca.* N. mikurensis”-positive nymphs were collected from the same location, and positive samples were therefore strongly correlated. One *I. ricinus* larva carried *B. garinii*, but the rest of the positive *B. burgdorferi* (*s.l.*) samples from nymphs that could be identified by sequencing were *B. afzelii*. The co-infection prevalence for juvenile ticks was 2.6% (4/152) for *I. ricinus* and 3.7% (1/27) for *I. persulcatus*. Three of the four positive “*Ca.* N. mikurensis” samples were co-infected with *B. afzelii*.

## Discussion

The distribution of *I. ricinus* and *I. persulcatus* in Finland is fairly unique [5, 29, 30]. The southern area of the country below the latitude 61°N is an area dominated by *I. ricinus*, while the area above 65°N is dominated by *I. persulcatus*. The belt between these latitudes is an area of sympatric occurrence of both species. In this study, we mapped the prevalence and distribution of an array of established and putative tick-borne pathogens in ticks in these three areas.

Overall, almost 30% of ticks were infected with at least one pathogen and 2% with more than one pathogen. Tick-borne pathogen diversity was higher in *I. ricinus* than in *I. persulcatus*. Of seven studied pathogen groups, five were detected in *I. ricinus* and three in *I. persulcatus*. A higher diversity of tick-borne pathogens in *I. ricinus* has also been observed in a previous study by Movila et al. [6], in which they investigated the differences of tick-borne microorganism communities in *I. ricinus* and *I. persulcatus* in distinct geographical regions of eastern Europe and European Russia. However, in our study, *I. ricinus* were also more frequently mono- and co-infected than *I. persulcatus*, which is contrary to the observations made by Movila et al. [6]. Infection rates can be influenced by many factors, e.g. life stage, sex, collection site of the ticks and by the season of tick collection. In Finland and neighboring countries, *I. persulcatus* adults have only one activity peak in April or May and are found to be questing only until July, while *I. ricinus* usually has two activity peaks during summer with the latter occurring during late August or September [5, 31, 32]. In our dataset, 85% of the *I. persulcatus* samples were collected by the end of May, while 85% of the *I. ricinus* samples were not collected until the end of July. Of all the analysed ticks, 95% were adults and less than one third were males, with these ratios equal in both species. Some of the studied pathogens in our study were analysed with single primers and some were multiplexed. Moreover, some were analysed individually while some were first pooled. These different methods could potentially cause a small bias on the prevalence of the studied pathogens. Nevertheless, our pathogen prevalence results mostly correspond with the prevalence results observed in the neighboring countries, suggesting that sample pooling and PCR multiplexing did not cause any major differences in the observed prevalences. However, slightly fewer positive findings could have been due to our “majority rules” approach when analyzing PCR results. Samples were considered positive when successful amplification was detected in at least two replicate reactions. A positive sample in one out of three replicates could suggest a low level of DNA close to the detection level rather than contamination of a sample. However, there were only a few samples that had only one positive replicate.

Not surprisingly, the most prevalent tick-borne pathogen was *B. burgdorferi* (*s.l.*) (17%), the causative agent of LB, which is the most prevalent tick-borne disease in Finland (around 6000–7000 cases yearly; ~120 cases per 100,000 inhabitants) [33]. The observed prevalence in our study is in accordance with the average prevalence rates found in Europe (17.8% for adult *I. ricinus* ticks) [34]. Even in the sympatric area, prevalence was significantly higher in *I. persulcatus* than *I. ricinus* (17.2 and 12.4%, respectively). A higher prevalence of *B. burgdorferi* (*s.l.*) in *I. persulcatus* than in *I. ricinus* ticks has also been observed in previous studies conducted in sympatric regions [30, 35, 36].

The most common *B. burgdorferi* (*s.l.*) genospecies detected in our study were *B. garinii* and *B. afzelii*. In comparison to reported *B. garinii* prevalence in *I. ricinus* ticks in Europe, a higher prevalence was observed in the present study [34]. Interestingly, a particularly high *B. garinii* prevalence (62.6% from *Borrelia*-positive ticks) was observed in *I. persulcatus* ticks. Migratory songbirds such as *Turdus* species are known as common *B. garinii* reservoir hosts while rodents are known as the main *B. afzelii* reservoir hosts [37, 38]. There can be differences in the occurrence of these reservoir species between countries, as well as fluctuations in their abundance (especially in voles) between different years [39], which might affect the proportions of *Borrelia* genospecies. Moreover, the activity peak of *I. persulcatus* in Finland co-occurs with the spring migration of *Turdus* spp. birds which might affect the higher *B. garinii* prevalence observed in *I. persulcatus*. *Borrelia afzelii* was observed relatively more often in *I. ricinus* than in *I. persulcatus* samples. The same observation was made in the study by Movila et al. [6]. While the evidence for human pathogenicity of *B. valaisiana* is poor, *B. afzelii*, *B. garinii* and *B. burgdorferi* (*s.s.*) are the genospecies that commonly infect people [4, 40]. These genospecies are also often associated with different clinical manifestations. In Europe, *B. garinii* is the main cause of Lyme neuroborreliosis, while *B. afzelii* is mostly associated with skin manifestations [41, 42]. Among the identified genospecies, *B. valaisiana* was detected in 6.4% of ticks, which is in accordance with the findings in the neighboring countries (6% in Norway, Sweden and Estonia) [30, 43, 44]. Even though, *B. valaisiana* is not often detected in *I. persulcatus*, it has been shown that in the sympatric areas of *I. ricinus* and *I. persulcatus*, *B. valaisiana* may exchange vectors and can also be found in *I. persulcatus* [30, 45]. In our study, *B. valaisiana* prevalence among *B. burgdorferi* (*s.l.*)-positive *I. persulcatus* samples (2.5%) was similar to the prevalence observed in Estonia, where these two tick species live in sympatric areas as well [30]. *B. burgdorferi* (*s.s.*) was not detected in *I. persulcatus* in this study, even though it has previously been found in *I. persulcatus* in a coastal Finnish region of the Gulf of Bothnia around the city of Kokkola [46].

Our study also revealed the occurrence of some less-known pathogens present in ticks in Finland. In contrast to *B. burgdorferi* (*s.l.*), all such pathogens had a higher prevalence in *I. ricinus* than in *I. persulcatus* samples. The most prevalent pathogen after *B. burgdorferi* (*s.l.*) spirochetes was *Rickettsia* spp. (10.8%). The observed prevalence is slightly higher than the reported prevalence in the neighboring country, Estonia (5.1%) [47]. Rickettsial DNA was more frequently detected in *I. ricinus* (13.9%) than in *I. persulcatus* (6.5%). The majority of the positive samples were identified as *R. helvetica* (90.9%). *Ixodes ricinus* is regarded as the main vector of *R. helvetica*, while the role of *I. persulcatus* is less studied. *Rickettsia helvetica* has been detected in *I. persulcatus* ticks before but with lower prevalence than in *I. ricinus* [6]. In our study, the prevalence of *R. helvetica* in *I. ricinus* (9.9%) was over four times higher than in *I. persulcatus* (2.2%), even though *R. helvetica* did not have a significant difference in infection prevalence between the collection areas. In contrast, “*Ca.* R. tarasevichae” was detected almost exclusively in *I. persulcatus* (18/19 positive samples). This is the first report of “*Ca.* R. tarasevichae” in Finland, and also the most western report. Moreover, to our knowledge this is the first report of the pathogen in *I. ricinus*. Interestingly, the positive *I. ricinus* sample is from southern Finland, the area of parapatric occurrence of *I. ricinus*. “*Ca.* R. tarasevichae” has previously been reported from neighboring countries Russia and Estonia [47, 48], where *I. persulcatus* is a common tick species. A minority (1.2%) of the *Rickettsia-*positive samples were identified as *R. monacensis*. This pathogen was first detected in *I. ricinus* ticks in 2013–2014 in southwestern Finland with similar prevalence to this study [18]. Patient cases from Spain and China suggest that “*Ca.* R. tarasevichae” and *R. monacensis* are both capable of human infection [49, 50].

*Anaplasma* spp. was the most abundant pathogen of the family *Anaplasmataceae* and almost all of the positive samples were detected in *I. ricinus*. The prevalence for *I. ricinus* was 1.0%, which is lower than the prevalence detected previously in questing *I. ricinus* adults in southwestern Finland (9.2%) [17]. However, reports from all over Europe have observed pronounced differences in prevalence among countries, study localities and tick life stages, ranging from 0 to 67% [9, 51, 52]. *Anaplasma phagocytophilum* is frequently detected in *I. ricinus* in Europe and it was the only *Anaplasma* species we detected. The prevalence for *I. persulcatus* was only 0.1% and neither of the two positive samples could be identified to species level by sequencing. *Anaplasma phagocytophilum* is the agent of human granulocytic anaplasmosis (HGA), and confirmed cases of HGA have been reported since 1997 in Europe [51, 53–55]. Another human pathogenic member of the family *Anaplasmataceae*, “*Ca.* N. mikurensis” was detected for the first time in ticks from mainland Finland, and only in *I. ricinus* (overall prevalence of 0.5%). The observed prevalence was lower than in previous observations made in Europe [56, 57]. The presence of “*Ca.* N. mikurensis” in *I. ricinus* ticks was previously analysed in ticks collected in 2013–2014 in southwestern Finland, but no positivity was detected [18]. However, our recent, still unpublished tick sampling data (Sormunen et al., in press), reveal the occurrence of this pathogen in southwestern Finland since 2015, agreeing with observations of the current study. “*Ca*. N. mikurensis” was also detected in *I. ricinus* ticks, collected in the years 2006–2013 in Estonia, with prevalence rates ranging from 1 to 9.1% [58].

Previous studies about *Babesia* spp. prevalence in Finnish ticks are scarce. The prevalence observed for *Babesia* spp. in *I. ricinus* samples in the current study (0.5%) corresponds to results reported from neighboring countries [30, 59–61]. *Babesia venatorum* was the most prevalent species in our study. In Europe and China this pathogen has been involved in the several documented cases of human babesiosis [62, 63]. *Babesia microti* was not detected, even though it is known to commonly infect rodents in Finland and was previously found in *I. persulcatus* tick in the Kokkola coastal region [46, 64]. The absence of *B. microti* in our study is likely due to low prevalence in Finnish ticks. *B. divergens* was detected in two samples. However, *B. divergens* is genetically very similar to *B. capreoli*. Since sequences should be identical to GenBank reference sequence U16370 to be regarded as *B. divergens* [65] and one of our positive samples were only 99% identical to this reference, we cannot be entirely sure whether this is truly *B. divergens* or *B. capreoli*. In Finland, a single case of fatal babesiosis in 2004 was caused by *B. divergens* and believed to be transmitted by a tick bite [19].

Neither *Bartonella* spp. nor *Francisella tularensis* were found in the ticks, although some *Bartonella* species have recently been reported from mammals and arthropods in Finland [66, 67]. The relevance of ticks as vectors for human bartonellosis is not yet verified. *Bartonella* have been reported in one *I. persulcatus* tick in Estonia (with a total prevalence of 0.2%) [6], while the prevalence in questing *I. ricinus* ticks in Europe has varied up to 48.2% in nymphs and 12% in adult ticks [68]. Although hard ticks are important vectors of *F. tularensis* in North America and central Europe [69–71], their role in the transmission of *F. tularensis* in northern Europe is poorly understood. In Fennoscandia, the primary route of human *F. tularensis* infection is probably through mosquito bites [69, 72–74].

In our study, about 7% of infected ticks were found to be co-infected. Most of the co-infections were between *B. burgdorferi* (*s.l.*) and *Rickettsia* spp., as expected due to their high prevalence. Interestingly, a particularly high number of co-infections were observed in positive “*Ca*. N. mikurensis” samples, of which over half (6/11) were co-infected with *B. burgdorferi* (*s.l.*) (Table 4). All of these co-infected *B. burgdorferi* (*s.l.*) samples were identified as *B. afzelii* and half of them were in nymph samples. “*Ca.* N. mikurensis” and *B. afzelii* share the same natural hosts (voles and mice) and co-infection with these pathogens has recently been shown to occur in nymphs more often than expected under random co-occurrence [57, 75, 76], as a result of larvae receiving both pathogens by feeding on co-infected hosts. The amount of different co-infection combinations was higher in *I. ricinus* (six) than in *I. persulcatus* (only one), partly due to the lower pathogen diversity and infection rates in *I. persulcatus* in our study. The co-infection rate in *I. ricinus* was 2.4%, which roughly corresponds with the results by Movila et al. (3.4%) [6]. However, in the same study, the co-infection rate in *I. persulcatus* samples was much higher than in ours (4.3 *vs* 0.8%). This is likely due to lower prevalence of *Rickettsia* infected *I. persulcatus* in our study. We examined co-infections only at genus level, and did not consider double infections among different pathogen species, e.g. *B. afzelii* and *B. garinii*. On average, 68% of the microbial sequences could be identified to species level. The rest of the sequence results were of poor quality (blurry or poorly resolved signal peaks in the chromatogram), which could have resulted from impurities in the DNA samples.

Species of *B. burgdorferi* (*s.l.*) and *Rickettsia* were detected beyond the latitude of 65°N, while the members of family *Anaplasmataceae* and *Babesia* spp. were detected only south of 65°N, perhaps due to lower abundance of *I. ricinus* in higher latitudes. The prevalence of infected ticks did not correlate directly with latitude, since the highest prevalence of infected ticks were found from the area below 61°N and from the area between 65°N and 66°N. Other ecological factors of the area, such as rainfall, distance to coast or inland waters, vegetation and variety of host animals, may have a bigger influence on pathogen diversity or proportion of the infected ticks [77]. According to previous observations, pathogen prevalence is also expected to correlate with the density of questing ticks at the collection sites [78–80]. Since our tick samples were gathered by citizens from all over Finland in a positive correlation to human population density, we cannot straightforwardly consider tick densities in our analyses. Interestingly, the highest infection rates were observed in areas of parapatric occurrence of one species, *I. ricinus* in southern and *I. persulcatus* in northern Finland. The assumption that in the zone of sympatry, the pathogen prevalence in one vector species would either increase or decrease by the influence of other closely related species, was not supported. Pathogen prevalence was lower for both species in the zone of their sympatry, indicating that other environmental factors might explain the lower prevalence in that area. When comparing infection rates between the collection areas by combining all the analysed ticks (excluding the information of tick species), the highest infection rate was observed in southern Finland, but infection rates did not differ between central and northern Finland (*I. ricinus*-dominated area: 32.6%; sympatric area: 25.4%; and *I. persulcatus*-dominated area: 25.9%). It remains unclear whether the higher prevalence in southern Finland is related to *I. ricinus* dominance or other environmental factors. *Rickettsia* spp. was the only pathogen group with a descending trend in infection prevalence towards the higher latitudes (*I. ricinus*-dominated area: 12.6%; sympatric area: 10.7%; and *I. persulcatus*-dominated area: 6.0%). However, the differences between the collection areas were not significant.

## Conclusions

The unique distribution of *I. ricinus* and *I. persulcatus* in Finland allowed us to compare the pathogen distributions in parapatric and sympatric areas of tick occurrence. The highest infection rate was observed in southern Finland, but infection rates did not differ between sympatric and *I. persulcatus-*dominated areas. However, the highest infection rates for both species were observed in areas of their dominance, either in southern or northern Finland. Furthermore, our comprehensive subset of Finnish ticks revealed the presence of rare and potentially pathogenic bacteria, such as “*Ca.* N. mikurensis” and “*Ca.* R. tarasevichae”, for the first time in mainland Finland. Although only a few human infections caused by organisms other than *B. burgdorferi* (*s.l.*) and TBEV have been reported in Finland so far, and it is known that infections due to these emerging pathogens can be either asymptomatic or mild [81, 82], the risk of these tick-borne pathogens for public health should not be neglected.

## Additional files

Additional file 1: **Methods.** Detailed protocols of qPCR assays and sequencing. (DOCX 25 kb)

Additional file 2: **Table S1.** Tick-borne pathogens in Finland: Comparison of *Ixodes ricinus* and *I. persulcatus* in sympatric and parapatric areas. (XLSX 13 kb)

## References

[1] Kernif T, Leulmi H, Raoult D, Parola P. Emerging tick-borne bacterial pathogens. Microbiol Spectr. 2016. 10.1128/microbiolspec.EI10-0012-2016. 10.1128/microbiolspec.EI10-0012-2016 27337487

[2] Dantas-Torres F, Chomel BB, Otranto D (2012). Ticks and tick-borne diseases: a One Health perspective. Trends Parasitol.

[3] Stanek G, Wormser GP, Gray J, Strle F (2012). Lyme borreliosis. Lancet.

[4] Rudenko N, Golovchenko M, Grubhoffer L, Oliver JH (2011). Updates on *Borrelia burgdorferi sensu lato* complex with respect to public health. Ticks Tick Borne Dis.

[5] Laaksonen M, Sajanti E, Sormunen JJ, Penttinen R, Hanninen J, Ruohomaki K (2017). Crowdsourcing-based nationwide tick collection reveals the distribution of *Ixodes ricinus* and *I. persulcatus* and associated pathogens in Finland. Emerg Microbes Infect.

[6] Movila A, Dubinina HV, Sitnicova N, Bespyatova L, Uspenskaia I, Efremova G (2014). Comparison of tick-borne microorganism communities in *Ixodes* spp. of the *Ixodes ricinus* species complex at distinct geographical regions. Exp Appl Acarol.

[7] Michelet L, Delannoy S, Devillers E, Umhang G, Aspan A, Juremalm M (2014). High-throughput screening of tick-borne pathogens in Europe. Front Cell Infect Microbiol.

[8] Rar V, Golovljova I (2011). *Anaplasma*, *Ehrlichia*, and “*Candidatus* Neoehrlichia” bacteria: pathogenicity, biodiversity, and molecular genetic characteristics, a review. Inf Genet Evol.

[9] Stuen S, Granquist EG, Silaghi C (2013). *Anaplasma phagocytophilum* - a widespread multi-host pathogen with highly adaptive strategies. Front Cell Infect Microbiol.

[10] Parola P, Paddock CD, Socolovschi C, Labruna MB, Mediannikov O, Kernif T (2013). Update on tick-borne rickettsioses around the world: a geographic approach. Clin Microbiol Rev.

[11] Platonov AE, Karan LS, Kolyasnikova NM, Makhneva NA, Toporkova MG, Maleev VV (2011). Humans infected with relapsing fever spirochete *Borrelia miyamotoi*, Russia. Emerg Inf Dis.

[12] Grankvist A, Andersson PO, Mattsson M, Sender M, Vaht K, Hoper L (2014). Infections with the tick-borne bacterium “*Candidatus* Neoehrlichia mikurensis” mimic noninfectious conditions in patients with B cell malignancies or autoimmune diseases. Clin Infect Dis.

[13] Homer MJ, Aguilar-Delfin I, Telford SR, Krause PJ, Persing DH (2000). Babesiosis. Clin Microbiol Rev.

[14] Petersen JM, Mead PS, Schriefer ME (2009). *Francisella tularensis*: an arthropod-borne pathogen. Vet Res.

[15] Billeter S, Levy M, Chomel B, Breitschwerdt E (2008). Vector transmission of *Bartonella* species with emphasis on the potential for tick transmission. Med Vet Entomol.

[16] Telford SR, Wormser GP (2010). *Bartonella* spp. transmission by ticks not established. Emerg Infect Dis.

[17] Sormunen JJ, Penttinen R, Klemola T, Vesterinen EJ, Hänninen J (2016). *Anaplasma phagocytophilum* in questing *Ixodes ricinus* ticks in southwestern Finland. Exp Appl Acarol.

[18] Sormunen JJ, Penttinen R, Klemola T, Hänninen J, Vuorinen I, Laaksonen M (2016). Tick-borne bacterial pathogens in southwestern Finland. Parasit Vectors.

[19] Haapasalo K, Suomalainen P, Sukura A, Siikamaki H, Jokiranta TS (2010). Fatal babesiosis in man, Finland, 2004. Emerg Infect Dis.

[20] Swanson SJ, Neitzel D, Reed KD, Belongia EA (2006). Coinfections acquired from *Ixodes* ticks. Clin Microbiol Rev.

[21] Belongia EA (2002). Epidemiology and impact of coinfections acquired from *Ixodes* ticks. Vector-Borne Zoonotic Dis.

[22] Moutailler S, Moro CV, Vaumourin E, Michelet L, Tran FH, Devillers E (2016). Co-infection of ticks: the rule rather than the exception. PLoS Negl Trop Dis.

[23] Diuk-Wasser MA, Vannier E, Krause PJ (2016). Coinfection by *Ixodes* tick-borne pathogens: ecological, epidemiological, and clinical consequences. Trends Parasitol.

[24] Thomas V, Anguita J, Barthold SW, Fikrig E (2001). Coinfection with *Borrelia burgdorferi* and the agent of human granulocytic ehrlichiosis alters murine immune responses, pathogen burden, and severity of Lyme arthritis. Infect Immun.

[25] Holden K, Hodzic E, Feng S, Freet KJ, Lefebvre RB, Barthold SW (2005). Coinfection with *Anaplasma phagocytophilum* alters *Borrelia burgdorferi* population distribution in C3H/HeN mice. Infect Immun.

[26] Rikihisa Y (2011). Mechanisms of obligatory intracellular infection with *Anaplasma phagocytophilum*. Clin Microbiol Rev.

[27] Sormunen JJ, Klemola T, Vesterinen EJ, Vuorinen I, Hytönen J, Hänninen J (2016). Assessing the abundance, seasonal questing activity, and *Borrelia* and tick-borne encephalitis virus (TBEV) prevalence of *Ixodes ricinus* ticks in a Lyme borreliosis endemic area in southwest Finland. Ticks Tick-Borne Dis.

[28] Coipan Elena Claudia, Fonville Manoj, Tijsse-Klasen Ellen, van der Giessen Joke W.B., Takken Willem, Sprong Hein, Takumi Katsuhisa (2013). Geodemographic analysis of Borrelia burgdorferi sensu lato using the 5S–23S rDNA spacer region. Infection, Genetics and Evolution.

[29] Bugmyrin SV, Bespyatova LA, Korotkov YS, Burenkova LA, Belova OA, Romanova LI (2013). Distribution of *Ixodes ricinus* and *I. persulcatus* ticks in southern Karelia (Russia). Ticks Tick-Borne Dis.

[30] Geller J, Nazarova L, Katargina O, Golovljova I (2013). *Borrelia burgdorferi sensu lato* prevalence in tick populations in Estonia. Parasit Vectors.

[31] Bormane Antra, Lucenko Irina, Duks Arnis, Mavtchoutko Violeta, Ranka Renate, Salmina Kristine, Baumanis Viesturs (2004). Vectors of tick-borne diseases and epidemiological situation in latvia in 1993–2002. International Journal of Medical Microbiology Supplements.

[32] Tokarevich N, Tronin A, Blinova O, Buzinov R, Boltenkov V, Yurasova E, Nurse J (2011). The impact of climate change on the expansion of *Ixodes persulcatus* habitat and the incidence of tickborne encephalitis in the north of European Russia. Global Health Action.

[33] Sajanti E, Virtanen M, Helve O, Kuusi M, Lyytikainen O, Hytonen J (2017). Lyme borreliosis in Finland, 1995–2014. Emerg Infect Dis.

[34] Strnad M, Honig V, Ruzek D, Grubhoffer L, Rego ROM (2017). Europe-wide meta-analysis of *Borrelia burgdorferi sensu lato* prevalence in questing *Ixodes ricinus* ticks. Appl Environ Microbiol.

[35] Kovalevskii YV, Korenberg EI (1995). Differences in *Borrelia* infections in adult *Ixodes persulcatus* and *Ixodes ricinus* ticks (Acari: Ixodidae) in populations of north-western Russia. Exp Appl Acarol.

[36] Alekseev AN, Dubinina HV, Antykova LP, Dzhivanyan TI, Rijpkema SG, Kruif NV (1998). Tick-borne borrelioses pathogen identification in *Ixodes* ticks (Acarina, Ixodidae) collected in St. Petersburg and Kaliningrad Baltic regions of Russia. J Med Entomol.

[37] Taragel'ova V., Koci J., Hanincova K., Kurtenbach K., Derdakova M., Ogden N. H., Literak I., Kocianova E., Labuda M. (2007). Blackbirds and Song Thrushes Constitute a Key Reservoir of Borrelia garinii, the Causative Agent of Borreliosis in Central Europe. Applied and Environmental Microbiology.

[38] Hanincova K, Schäfer S, Etti S, Sewell H, Taragelova V, Ziak D (2003). Association of *Borrelia afzelii* with rodents in Europe. Parasitology.

[39] Hansson L, Henttonen H (1985). Gradients in density variations of small rodents: the importance of latitude and snow cover. Oecologia.

[40] Margos G, Sing A, Fingerle V (2017). Published data do not support the notion that *Borrelia valaisiana* is human pathogenic. Infection.

[41] Strle F, Ružić-Sabljić E, Cimperman J, Lotrič-Furlan S, Maraspin V (2006). Comparison of findings for patients with *Borrelia garinii* and *Borrelia afzelii* isolated from cerebrospinal fluid. Clin Inf Dis.

[42] Jahfari S, Krawczyk A, Coipan EC, Fonville M, Hovius JW, Sprong H (2017). Enzootic origins for clinical manifestations of Lyme borreliosis. Inf Genet Evol.

[43] Jenkins A, Hvidsten D, Matussek A, Lindgren P, Stuen S, Kristiansen B (2012). *Borrelia burgdorferi sensu lato* in *Ixodes ricinus* ticks from Norway: evaluation of a PCR test targeting the chromosomal flaB gene. Exp Appl Acarol.

[44] Fraenkel CJ, Garpmo U, Berglund J (2002). Determination of novel *Borrelia* genospecies in Swedish *Ixodes ricinus* ticks. J Clin Microbiol.

[45] Alekseev AN, Dubinina HV, Van De Pol I, Schouls LM (2001). Identification of *Ehrlichia* spp. and *Borrelia burgdorferi* in *Ixodes ticks* in the Baltic regions of Russia. J Clin Microbiol.

[46] Alekseev AN, Dubinina HV, Jääskeläinen AE, Vapalahti O, Vaheri A (2007). First report on tick-borne pathogens and exoskeletal anomalies in *Ixodes persulcatus* schulze (Acari: Ixodidae) collected in Kokkola coastal region, Finland. Int J Acarol.

[47] Katargina O, Geller J, Ivanova A, Värv K, Tefanova V, Vene S (2015). Detection and identification of *Rickettsia* species in *Ixodes* tick populations from Estonia. Ticks Tick-Borne Dis.

[48] Shpynov S, Fournier P, Rudakov N, Raoult D (2003). ‘*Candidatus* Rickettsia tarasevichiae’ in *Ixodes persulcatus* ticks collected in Russia. Ann N Y Acad Sci.

[49] Jia N, Zheng Y, Jiang J, Ma L, Cao W (2013). Human infection with *Candidatus* Rickettsia tarasevichiae. N Engl J Med.

[50] Jado I, Oteo JA, Aldamiz M, Gil H, Escudero R, Ibarra V (2007). *Rickettsia monacensis* and human disease, Spain. Emerg Infect Dis.

[51] Blanco J, Oteo J (2002). Human granulocytic ehrlichiosis in Europe. Clin Microbiol Inf.

[52] Strle Franc (2004). Human granulocytic ehrlichiosis in Europe. International Journal of Medical Microbiology Supplements.

[53] Brouqui P, Dumler J, Lienhard R, Brossard M, Raoult D (1995). Human granulocytic ehrlichiosis in Europe. Lancet.

[54] Edouard S, Koebel C, Goehringer F, Socolovschi C, Jaulhac B, Raoult D (2012). Emergence of human granulocytic anaplasmosis in France. Ticks Tick-Borne Dis.

[55] Petrovec M, Lotric Furlan S, Zupanc TA, Strle F, Brouqui P, Roux V (1997). Human disease in Europe caused by a granulocytic *Ehrlichia* species. J Clin Microbiol.

[56] Derdáková M, Václav R, Pangrácova-Blaňárová L, Selyemová D, Koči J, Walder G (2014). *Candidatus* Neoehrlichia mikurensis and its co-circulation with *Anaplasma phagocytophilum* in *Ixodes ricinus* ticks across ecologically different habitats of central Europe. Parasit Vectors.

[57] Kjelland V, Paulsen KM, Rollum R, Jenkins A, Stuen S, Soleng A (2018). Tick-borne encephalitis virus, *Borrelia burgdorferi sensu lato*, *Borrelia miyamotoi*, *Anaplasma phagocytophilum* and *Candidatus* Neoehrlichia mikurensis in *Ixodes ricinus* ticks collected from recreational islands in southern Norway. Ticks Tick-Borne Dis.

[58] Ivanova A, Geller J, Katargina O, Värv K, Lundkvist Å, Golovljova I (2017). Detection of *Candidatus* Neoehrlichia mikurensis and *Ehrlichia muris* in Estonian ticks. Ticks Tick-Borne Dis.

[59] Katargina O, Geller J, Vasilenko V, Kuznetsova T, Järvekülg L, Vene S (2011). Detection and characterization of *Babesia* species in *Ixodes* ticks in Estonia. Vector-Borne Zoon Dis.

[60] Karlsson ME, Andersson MO (2016). *Babesia* species in questing *Ixodes ricinus*, Sweden. Ticks Tick-Borne Dis.

[61] Rar V, Epikhina T, Livanova N, Panov V (2011). Genetic diversity of *Babesia* in *Ixodes persulcatus* and small mammals from North Ural and West Siberia, Russia. Parasitology.

[62] Jiang J, Zheng Y, Jiang R, Li H, Huo Q, Jiang B (2015). Epidemiological, clinical, and laboratory characteristics of 48 cases of “*Babesia venatorum*” infection in China: a descriptive study. Lancet Inf Dis.

[63] Hunfeld K, Hildebrandt A, Gray J (2008). Babesiosis: recent insights into an ancient disease. Int J Parasitol.

[64] Kallio ER, Begon M, Birtles RJ, Bown KJ, Koskela E, Mappes T (2014). First report of *Anaplasma phagocytophilum* and *Babesia microti* in rodents in Finland. Vector-Borne Zoon Dis.

[65] Malandrin L, Jouglin M, Sun Y, Brisseau N, Chauvin A (2010). Redescription of *Babesia capreoli* (Enigk and Friedhoff, 1962) from roe deer (*Capreolus capreolus*): isolation, cultivation, host specificity, molecular characterisation and differentiation from *Babesia divergens*. Int J Parasitol.

[66] Veikkolainen V, Vesterinen EJ, Lilley TM, Pulliainen AT (2014). Bats as reservoir hosts of human bacterial pathogen, *Bartonella mayotimonensis*. Emerg Infect Dis.

[67] Lilley TM, Veikkolainen V, Pulliainen AT (2015). Molecular detection of *Candidatus* Bartonella hemsundetiensis in bats. Vector-Borne Zoon Dis.

[68] Dietrich F, Schmidgen T, Maggi RG, Richter D, Matuschka FR, Vonthein R (2010). Prevalence of *Bartonella henselae* and *Borrelia burgdorferi sensu lato* DNA in *Ixodes ricinus* ticks in Europe. Appl Environ Microbiol.

[69] Ryden P, Bjork R, Schafer ML, Lundstrom JO, Petersen B, Lindblom A (2012). Outbreaks of tularemia in a boreal forest region depends on mosquito prevalence. J Infect Dis.

[70] KEIM P., JOHANSSON A., WAGNER D. M. (2007). Molecular Epidemiology, Evolution, and Ecology of Francisella. Annals of the New York Academy of Sciences.

[71] Eisen RJ, Mead PS, Meyer AM, Pfaff LE, Bradley KK, Eisen L (2008). Ecoepidemiology of tularemia in the southcentral United States. Am J Trop Med Hyg.

[72] Eliasson H, Lindbäck J, Nuorti P, Arneborn M, Giesecke J, Tegnell A (2002). The 2000 tularemia outbreak: a case-control study of risk factors in disease-endemic and emergent areas, Sweden. Emerg Inf Dis.

[73] Rossow H, Ollgren J, Klemets P, Pietarinen I, Saikku J, Pekkanen E (2014). Risk factors for pneumonic and ulceroglandular tularaemia in Finland: a population-based case-control study. Epidemiol Infect.

[74] Thelaus J, Andersson A, Broman T, Bäckman S, Granberg M, Karlsson L (2014). *Francisella tularensis* subspecies *holarctica* occurs in Swedish mosquitoes, persists through the developmental stages of laboratory-infected mosquitoes and is transmissible during blood feeding. Microb Ecol.

[75] Andersson M, Bartkova S, Lindestad O, Råberg L (2013). Co-infection with ‘*Candidatus* Neoehrlichia mikurensis’ and *Borrelia afzelii* in *Ixodes ricinus* ticks in southern Sweden. Vector-Borne Zoon Dis.

[76] Richter D, Matuschka FR (2012). “*Candidatus* Neoehrlichia mikurensis”, *Anaplasma phagocytophilum*, and Lyme disease spirochetes in questing european vector ticks and in feeding ticks removed from people. J Clin Microbiol.

[77] Coipan EC, Jahfari S, Fonville M, Maassen CB, van der Giessen J, Takken W (2013). Spatiotemporal dynamics of emerging pathogens in questing *Ixodes ricinus*. Front Cell Infect Microbiol.

[78] Randolph SE (2001). The shifting landscape of tick-borne zoonoses: tick-borne encephalitis and Lyme borreliosis in Europe. Philos Trans R Soc Lond B Biol Sci.

[79] Ogden N (2013). Changing geographic ranges of ticks and tick-borne pathogens: drivers, mechanisms and consequences for pathogen diversity. Front Cell Inf Microbiol.

[80] Ogden N, Casey A, French N, Adams J, Woldehiwet Z (2002). Field evidence for density-dependent facilitation amongst *Ixodes ricinus* ticks feeding on sheep. Parasitology.

[81] Ismail N, Bloch KC, McBride JW (2010). Human ehrlichiosis and anaplasmosis. Clin Lab Med.

[82] Tijsse-Klasen E, Jacobs JJ, Swart A, Fonville M, Reimerink JH, Brandenburg AH (2011). Small risk of developing symptomatic tick-borne diseases following a tick bite in The Netherlands. Parasit Vectors.

[83] Ivacic L, Reed KD, Mitchell PD, Ghebranious N (2007). A LightCycler TaqMan assay for detection of *Borrelia burgdorferi sensu lato* in clinical samples. Diagn Microbiol Infect Dis.

[84] Labruna MB, Whitworth T, Horta MC, Bouyer DH, McBride JW, Pinter A (2004). *Rickettsia* species infecting *Amblyomma cooperi* ticks from an area in the state of Sao Paulo, Brazil, where Brazilian spotted fever is endemic. J Clin Microbiol.

[85] Øines Ø, Radzijevskaja J, Paulauskas A, Rosef O (2012). Prevalence and diversity of *Babesia* spp. in questing *Ixodes ricinus* ticks from Norway. Parasit Vectors.

[86] Courtney JW, Kostelnik LM, Zeidner NS, Massung RF (2004). Multiplex real-time PCR for detection of *Anaplasma phagocytophilum* and *Borrelia burgdorferi*. J Clin Microbiol.

[87] Jahfari S, Fonville M, Hengeveld P, Reusken C, Scholte E, Takken W (2012). Prevalence of *Neoehrlichia mikurensis* in ticks and rodents from North-west Europe. Parasit Vectors.

[88] Diaz MH, Bai Y, Malania L, Winchell JM, Kosoy MY (2012). Development of a novel genus-specific real-time PCR assay for detection and differentiation of *Bartonella* species and genotypes. J Clin Microbiol.

[89] Skottman T, Piiparinen H, Hyytiäinen H, Myllys V, Skurnik M, Nikkari S (2007). Simultaneous real-time PCR detection of *Bacillus anthracis*, *Francisella tularensis* and *Yersinia pestis*. Eur J Clin Microbiol Inf Dis.

[90] Mediannikov O, Sidelnikov Y, Ivanov L, Mokretsova E, Fournier P, Tarasevich I, et al. New acute tick-borne rickettsiosis caused by *Rickettsia heilongjiangensis* in the Russian Far East. Clin Microbiol Inf. 2004;10(Suppl.):7–8. 10.3201/eid1005.030437 PMC3323216 15200813

[91] Hildebrandt A, Krämer A, Sachse S, Straube E (2010). Detection of *Rickettsia* spp. and *Anaplasma phagocytophilum* in *Ixodes ricinus* ticks in a region of Middle Germany (Thuringia). Ticks Tick-Borne Dis.

[92] Georges K, Loria G, Riili S, Greco A, Caracappa S, Jongejan F (2001). Detection of haemoparasites in cattle by reverse line blot hybridisation with a note on the distribution of ticks in Sicily. Vet Parasitol.

