# Supplementary material for: Tick-borne pathogens in Finland: comparison of Ixodes ricinus and I. persulcatus in sympatric and parapatric areas
Source: Parasit Vectors. 2018 Oct 24;11:556. doi: 10.1186/s13071-018-3131-y (PMC6201636; doi:10.1186/s13071-018-3131-y)
Supplement: Supplementary file 1 — Methods. Detailed protocols of qPCR assays and sequencing. (DOCX 25 kb) [file 13071_2018_3131_MOESM1_ESM.docx]

**Additional file 1. Methods**

**Real-time quantitative PCR assays**

Bbsl-ospA-F and Bbsl-ospA-R primers and a Bbsl-ospA-P probe (Table 1), amplifying a 102-bp fragment of the outer surface protein A (ospA) gene, were used to detect *B. burgdorferi* (*s.l*.) DNA as previously described [1]. Positive and negative controls (*B. burgdorferi* (*sensu* *stricto*) strain B31 ATCC 35210 and ddH2O, respectively) were included in all runs. The amplification of ospA gene was carried out in a final reaction volume of 20 µl, containing 600 nM primers (Bbsl-ospA-F and Bbsl-ospA-R) and 100 nM probe (Bbsl-ospA-P), 10 µl of LightCycler 480 Probes Master (Roche Diagnostics GmbH, Mannheim, Germany), 5 µl of ddH2O, and 2 µl of template DNA. The thermal cycling was performed under the following conditions: 95°C for 10 min, 55 cycles of 95°C for 10 s and 58°C for 1 min, followed by cooling at 40°C for 1 min.

For *Anaplasma*, *Babesia* and "*Candidatus* Neoehrlichia mikurensis" screening, multiplex qPCR were used to save time and reagents. Briefly, qPCRs were performed in 11-μl volumes using the SensiFAST Probe Lo-ROX Kit (BIO-84020, Bioline Reagents Ltd, UK). Fragments of the 18S rDNA, surface protein antigen Msp2, and the chaperonin GroEL were amplified from tick lysates using the primers and probes displayed in Table 1. Assays were carried out in 11 μl reaction volume, including 5,5 μl SensiFAST Probe Lo-ROX Kit, 0.75 μl ddH2O, 400 nM primer ApMsp2_F+R Mix, 200 nM primer CNeGroEL_F+R Mix, 400 nM primer Bab18S_F+R Mix, 200 nM probe ApMsp2, 100 nM probe CNeGroEL, 200 nM probe Bab18S and 2 μl of pooled DNA. DNA samples of *A. phagocytophilum* extracted from the blood of infected voles provided by Eva R. Kallio (University of Oulu) were used as positive controls. DNA samples of *Babesia* and "*Ca*. N. mikurensis" confirmed by sequencing in an earlier study were used as positive controls as well.

For *Bartonella* and *Rickettsia* we used a duplex qPCR assay with primers targeting *Bartonella* ssrA (Bart-ssRA-F and Bart-ssRA-R) and *Rickettsia* gltA (Rspp-F and Rspp-R), and dual-labeled probes Bart ssRA- P and Rspp-P. Assays were carried out in 8 μl reaction volume, including 4 μl SensiFAST Probe Lo-ROX Kit, 200 nM Bartonella forward and reverse primers, 300 nM Rickettsia forward and reverse primers, 100 nM Bartonella probe, 150 nM Rickettsia probe, and 3 μl of pooled DNA sample. As positive controls for *Bartonella*, we used patient strains of *B. grahamii* and *B. quintana*, provided by Arto Pulliainen (University of Turku). For *Rickettsia*, we used a commercially available control sample (MBC042, Vircell, Granada, Spain).

Primers Ftu23-F and Ftu23-R and Ftu23-P probe targeting 23 KDa gene were used to detect *Francisella tularensis* DNA. Positive and negative controls (MBC110; Vircell, Granada, Spain and ddH2O, respectively) were included in all runs. Assays were carried out in 5 μl reaction volume, including 2,5 μl SensiFAST Probe Lo-ROX Kit, 300 nM FTU23 forward and reverse primers, 150 nM probe and 1 μl DNA.

**Sequencing**

Samples found positive for *Rickettsia* spp. were sequenced using conventional PCR primers (CS877f and CS1258r; Table 1) targeting *Rickettsia* gltA gene. PCR was carried out in 10 μl reaction volume containing 3 μl of DNA extract, 1.5 μl ddH2O, 5 μl MyTaq Red Mix polymerase mix (product number BIO-25048, Bioline, England), 250 nM forward primer, and 250 nM reverse primer. Thermal cycling was performed with the following program: 95 °C for 3 min, then 50 cycles of 95 °C for 20 s, 64 °C for 30 s, and 72 °C for 1 min.

Samples found positive for *Anaplasma* spp. by qPCR were sequenced using PCR primers (Ana2f and Ana2r; Table 1) targeting *Anaplasma* 16S rRNA gene. PCR was carried out in 10 μl reaction volume containing 3 μl of DNA extract, 1.5 μl ddH2O, 5 μl MyTaq Red Mix polymerase mix, 400 nM forward and reverse primers. Thermal cycling was performed with the following program: 95 °C for 3 min, then 50 cycles of 95 °C for 30 s, 62 °C for 20 s, and 72 °C for 1 min.

Samples found positive for *Babesia* spp. by qPCR were sequenced using PCR primers (BabNu2f and BabNu2r; Table 1) targeting *Babesia* 18S rRNA gene. PCR was carried out in 10 μl reaction volume containing 3 μl of DNA extract, 1.5 μl ddH2O, 5 μl MyTaq Red Mix polymerase mix, 400 nM forward and reverse primers. Thermal cycling was performed with the following program: 95 °C for 3 min, then 50 cycles of 95 °C for 30 s, 58 °C for 20 s, and 72 °C for 1 min.

**Reference**

1. Ivacic L, Reed KD, Mitchell PD, Ghebranious N. A LightCycler TaqMan assay for detection of Borrelia burgdorferi sensu lato in clinical samples. Diagn Microbiol Infect Dis. 2007;57(2):137-43.
